# Microbial characteristics of dental caries in HIV positive individuals

**DOI:** 10.3389/froh.2022.1004930

**Published:** 2022-09-21

**Authors:** Dunstan Kalanzi, Harriet Mayanja-Kizza, Damalie Nakanjako, Fred Semitala, Gerald Mboowa, Muhammad Mbabali, Edgar Kigozi, Fred Ashaba Katabazi, Ivan Sserwadda, David P. Kateete, Beatrice Achan, Nelson K. Sewankambo, Adrian Muwonge

**Affiliations:** ^1^ Department of Dentistry, School of Health Sciences, Makerere University College of Health Sciences, Kampala, Uganda; ^2^ Department of Medicine, School of Medicine, Makerere University College of Health Sciences, Kampala, Uganda; ^3^ Department of Immunology and Molecular Biology, School of Biomedical Sciences, Makerere University College of Health Sciences, Kampala, Uganda; ^4^ Department of Medical Microbiology, School of Biomedical Sciences, Makerere University College of Health Sciences, Kampala, Uganda; ^5^ Division of Genetics and Genomics, The Roslin Institute, University of Edinburgh, Edinburgh, United Kingdom

**Keywords:** human immunodeficiency virus, microbial co-occurrence networks, dental caries, oral microbiota, DMFT index

## Abstract

**Background:**

Dental caries is a multifactorial disease that affects many people. Even though microorganisms play a crucial role in causing dental caries, diagnosis is routinely macroscopic. In order to improve early detection especially in HIV patients who are disproportionately affected, there is need to reconcile the macroscopic and microscopic characteristics of dental caries. Therefore, the aim of this study was to characterize the oral microbiota profile along the decayed, missing, filled teeth (DMFT) index using amplicon sequencing data.

**Methods:**

Amplicon sequencing of the V6-V8 region of the *16S rRNA* gene was done on DNA recovered from whole unstimulated saliva of 59 HIV positive and 29 HIV negative individuals. The microbial structure, composition and co-occurrence networks were characterized using QIIME-2, Phyloseq, Microbiome-1.9.2 and Metacoder in R.

**Results:**

We characterized the oral microbiota into 2,093 operational taxonomic units (OTUs), 21 phyla and 239 genera from 2.6 million high quality sequence reads. While oral microbiota did not cluster participants into distinct groups that track with the DMFT index, we observed the following: (a) The proportion of accessory microbiota was highest in the high DMFT category while the core size (∼50% of richness) remained relatively stable across all categories. (b) The abundance of core genera such as *Stomatobaculum*, *Peptostreptococcus* and *Campylobacter* was high at onset of dental caries, (c) A general difference in oral microbial biomass. (d) The onset of dental caries (low DMFT) was associated with significantly lower oral microbial entropy.

**Conclusions:**

Although oral microbial shifts along the DMFT index were not distinct, we demonstrated the potential utility of microbiota dynamics to characterize oral disease. Therefore, we propose a microbial framework using the DMFT index to better understand dental caries among HIV positive people in resource limited settings.

## Introduction

Dental caries is a highly prevalent infectious disease that affects a third of the world's population (1). Dental caries is caused by a number of factors, such as, consumption of refined sugars, inadequate dental hygiene, susceptible tooth surfaces, microorganisms, and time (2). Dental caries is normally diagnosed by visual inspection; and in oral health surveys, it is recorded using the decayed, missing (due to caries) and filled teeth (DMFT) index (3). The DMFT index is a simple and common macroscopic tool (4).

Although dental caries diagnosis is normally based on visual inspection, microorganisms play a significant role in causing caries (5). With the advent of next-generation sequencing (NGS) technology, it is now possible to study the intricate relationships that exist in microbial communities (6), including those found in the oral cavity. Furthermore, the application of this technology in medicine, clinical metagenomics NGS (mNGS), is one of the fastest growing areas of medicine in the developed world (7).

While characterizing oral diseases such as dental caries through oral microbiota is common in the developed world, it remains largely unexplored in developing countries. This is of particular concern because many developing countries have a high burden of disease, which includes HIV. Moreover, it has been reported that people living with HIV/AIDS are at a higher risk of developing dental caries compared to the general population (8, 9). For example, in Uganda, 1.3 million people are infected with HIV (10), and the prevalence of dental caries in this patient population was found to be 83.7% with a mean DMFT of 5.9 (11). This is higher than the prevalence of dental caries in the general adult population reported to be 66.7% with a mean DMFT of 4.71 (12). However, the robust tracking of microbial changes based on the severity of dental caries in people living with HIV has paucity of data.

To address this paucity of research on tracking microbial characteristics in people living with HIV, we characterized oral microbiota differences along the DMFT index in HIV positive and negative individuals using 16S rRNA gene sequencing in a resource limited setting, as a stepping stone towards the application of clinical metagenomics in oral health.

## Materials and methods

### Ethical considerations

The School of Medicine Research and Ethics committee of Makerere University (#REC REF 2017-053) approved this study, which was conducted in accordance with the ethical standards outlined in the 1964 Declaration of Helsinki and its later amendments. The research team obtained written informed consent from all participants recruited for the study.

### Study participants and setting

This was a cross sectional study carried out at the Mulago Immune Suppression Syndrome (ISS) clinic, which is an HIV care clinic under the Makerere University Joint AIDS Program (MJAP). The Mulago ISS clinic has been in operation for over 15 years and provides HIV related services to approximately 16,000 patients annually, 80% of whom are on anti-retroviral treatment (ART). The clinicians treat on average 300 people every day, which makes it one of few places where HIV related co-morbidities like dental disease can be studied. For this study, we recruited and sampled HIV positive and negative adults at this clinic.

### Sample size determination

We conveniently sampled 88 persons of the 168 who participated in the study between January and May 2018. This is because only a few studies have reported on oral clinical metagenomics in low and middle income countries (LMICs); therefore, information on powering such studies is limited (13). Studies conducted elsewhere have used 50–65 samples to detect up to 80% of genera (14, 15).

### Sample collection

We collected saliva samples for oral microbiome analysis between 9:00 AM and 12:00 noon, in order to minimize the effect of the circadian rhythm, according to a published protocol (16). For measurement of flow rate, saliva was collected for 5 min without any stimulation. Participants were asked not to swallow, but to expel the accumulated saliva into a calibrated plastic centrifuge tube at intervals over a period of 5 min. Saliva samples were collected on ice, transported on ice, and stored at −80°C prior to component analysis. Trained clinicians performed the oral examinations using dental probes and mirrors under suitable artificial light after saliva collection at the same visit.

### Clinical characterization of dental caries

**Figure 1:**
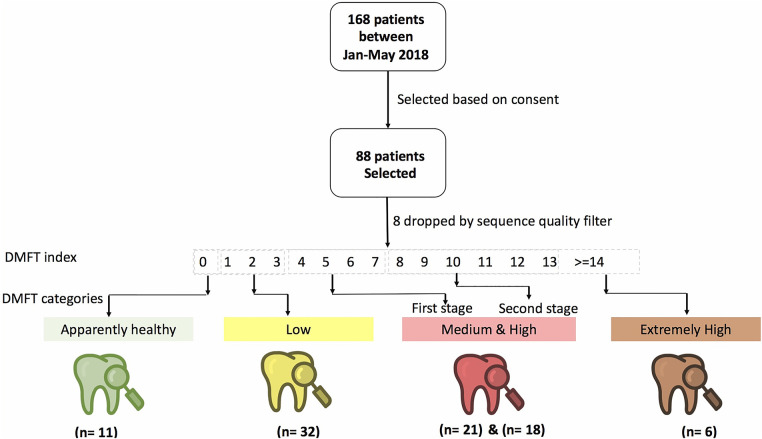
Shows the study design, patient and DMFT categorization of participants recruited from the Mulago Immune Suppression Syndrome (ISS) clinic at Makerere University Joint AIDS Program.

We assessed caries status using the World Health Organization (WHO) caries classification criteria and reported these findings using the decayed (D), missing (M), filled (F), teeth (DMFT) index (17). A DMFT score of >0 was considered as dental caries and thereafter, we categorized the participants into healthy (DMFT = 0), low (DMFT = 1–3), medium (DMFT = 4–6), high (DMFT = 7–13) and extremely high (DMFT > 14) (Figure 1).

### Periodontal health status

Periodontal health was assessed using a minimum diagnostic criteria of bleeding on probing and periodontal pocket depth measurements. Participants with only bleeding on probing were considered to have gingivitis, while those with periodontal pockets of 4 mm and greater were considered to have some form of periodontitis (18).

### Saliva processing and DNA extraction

Saliva samples were processed as follows prior to DNA extraction using GenoLyse method. While in a biosafety cabinet, 3 milliliters of saliva were transferred to 15 ml sterile centrifuge tubes, and an equal volume of 1% w/v NaCl was added. The samples were vortexed vigorously for 1 min until the specimen was fully liquefied. This was done in order to digest saliva to release the bacteria. Phosphate buffered saline (PBS) pH 6.8 was then added up to the 15 ml mark. The specimens were then centrifuged at 3,000 × g for 15 min at 4 °C, and the supernatant discarded. The resulting pellet was suspended into 1 ml of 1XPBS and then transferred to a 1.5 ml nuclease free centrifuge tube. These were then centrifuged at 10,000 × g for 15 min prior to extraction of DNA using the GenoLyse method, following manufacturer's recommendations (Bruker, USA). Briefly, the supernatant was discarded and the pellet resuspended in 100 mls of GenoLyse lysis buffer (A-LYS) prior to gentle vortexing to lyse the cells of the microorganisms including bacteria. Additional cell lysis was achieved by incubating the tubes at 95 °C for 5 min prior to adding 100 mls of GenoLyse neutralizing buffer (A-NB) to stop the action of A-LYS. The mixture was vortexed again for about 2 s prior to centrifugation at 13,000 rpm for 5 min. DNA was collected from the supernatant and stored prior to use in subsequent analyses.

### 16S rRNA gene sequencing

Aliquots of 30 µl were then shipped under controlled ambient condition to Dalhousie University Integrated Microbiome Resource (IMR, Canada) (19). Following purification of the amplicon pools using AMPure beads, sequencing of the V6-V8 16S rRNA variable loops was performed on the Illumina MiSeq platform (San Diego, CA, USA) using the 400 paired-end MiSeq run according to an established protocol (20). Following sequencing, demultiplexed samples were returned.

### Sequence analysis

We received a total of 88 paired end sequences in fastq format *via* an html file transfer link from the Dalhousie University Integrated Microbiome Resource (IMR) (19). Demultiplexed reads from the sequencing facility were then imported into the QIIME 2 pipeline (21) for analysis. As part of quality control, the paired-ended reads were trimmed and merged into single ended reads. A QIIME artifact was then generated from these sequences and the metadata file. After dereplication, chimera-removal and denoising using DADA2 (21), an OTUs dataset was generated. We filtered OTUs with a sequence depth of at least 3,000, and retained 90% of the samples and the corresponding metadata (22).

### Microbial community structure

We used QIIME 2 and R-based packages; Phyloseq and Microbiome analyzer for microbial diversity analysis. The OTU-data output was used to estimate the Alpha and Beta diversity indices. We considered observed Shannon for the Alpha diversity analysis (23), whereas Beta diversity was estimated with Bray-Curtis, Constrained analysis of principal coordinates (CAP), Jensen-Shannon divergence (JSD), weighted and unweighted Unifrac distances (21). To examine the association of clinical variables on the oral microbiota structure, we used a permutational multivariate analysis of variances (PERMANOVA) with the Adonis function (9,999 permutations) in phyloseq (23) using the estimated Beta diversity indices as the outcome variable. The results were converted into a bar plot ranking the effect size R^2^ of each clinical variable and its statistical significance. To examine if participants can be clustered using beta diversity indices, we run CAP, JSD and Bray-Curtis indices. The analysis was constrained by DMFT and periodontal health status (gingivitis and or periodontitis). We also run a principal coordinate analysis (PCoA) of weighted unifrac distance, which was used to estimate the total variance explained by the first five components, henceforth referred to as TVE (24) in Phyloseq. The impact of genera on TVE was used to evaluate the most influential microbes in each DMFT category.

### Parameters for characterizing DMFT

In order to map and track microbial differences along the DMFT index, we characterized changes in the alpha and beta diversity indices, abundance and prevalence, and co-occurrence dynamics.

### Microbial composition

To investigate differences in abundance, we used Metacoder as described by Foster (25) which combines phylogenetics and abundance. This allowed us to track the different abundant genera along the DMFT index. Briefly, this analysis was done using a Metacoder object generated from QIIME 2 taxonomic classification of OTUs using a naïve Bayes classifier trained on the most recent SILVA database at 97% similarity (26). First, a training dataset was extracted using the primers used for sequencing our samples. The resultant database subset was then used to train the classifier for taxonomically assigning the OTUs. The heat tree highlights branches based on abundance. To determine the influential genera in DMFT categories, we ranked the fifty most abundant genera, and then examined their impact on TVE by sequentially removing them from the data set representing each DMFT category.

### Core and accessory microbiota

To understand core and accessory differences as a proportion of the total richness along the DMFT index, first, we defined the core as the OTUs present in 85% of the samples at each DMFT category. Secondly, the pan-microbiota was taken as the total number of unique OTUs in each category, and the accessory computed as the difference between the pan and core microbiota in each category. We then analyzed the data to detect these microbial components in our samples using microbiome package 1.9.2 (27), the output was summarized and plotted using Tidyrverse 1.2.1 and ggplot2 2.3.1 in R, respectively. To track the core as a proportion of microbial richness, we selected genera shared by all participants in all the DMFT categories then divided that by the richness at each stage. To examine the influence of genera, we first ranked nodes (genera) in the co-occurrence network by their centrality degree after which, we selected the top 50 genera (see co-occurrence network) and then examined their impact on TVE. This was done by sequentially dropping one genus from the selected genera and computing the change in TVE. The change in variance was visualized using bar plots in ggplot colored by the oxygen utilization capacity of each genus. We used the term invaders interchangeably with accessory microbiota, as they represented transient genera. We also used published literature elsewhere to create two other categories i.e., oral-disease associated genera and normal flora (28, 29) (see detailed lists in the Supplementary).

### Microbial co-occurrence networks

We used the mean abundance correlation matrix of the genera in each DMFT category to map the differences in the co-occurrence network (30, 31). The “associate” function within the microbiome package version 1.9.2 in R (v3.5.1) was used to generate a genus-level spearman correlation matrix, here we set the FDR adjusted *p*-value at 0.05 (32) and pruned the matrix using a correlation coefficient of >0.5 and <–0.5. The resultant matrix was then converted into a directed-network object from which communities were extracted and visualized in igraph package version 1.2.2. The edges were colored based on statistical significance (Supplementary Figure. S6).

### A quasi-Poisson logistic regression for the DMFT

To determine the factors associated with differences in biomass along the DMFT index, we developed a Poisson regression model in GLM where the outcome variable was mean taxonomic abundance (microbial biomass) using the lme4 package in R version (v3.5.1). We split the data into five subsets each representing a DMFT category, and then run five separate models with the same explanatory variables. It is these models we compared to examine the difference in estimates; the differences here represented the changes at each stage accounting for gender, HIV status and microbes at family level. Model comparison was done using sjtools in in R (v3.5.1).

## Results

### Participants descriptive summary

This study involved 38 and 50 male and female participants (*N* = 88) with an average age of 39.5 years. The proportion of HIV positive participants was 67% (59/88) with a median CD4 T cell count of 402 cells/mm^3^, and a viral load of ≤50 copies/ml of blood. All HIV positive participants were on ART. The mean salivary flow rate was 0.9 ml/min with no discernable difference between HIV positive and HIV negative participants. We observed a difference in the mean DMFT among HIV negative and positive participants of 5.9 and 4.9, respectively (Table 1).

**Table 1:** Descriptive statistics of samples.

	Number (%)	HIV status	
		HIV+	HIV–
		59 (67.1)	29 (32.9)
Sex			
Female	50 (56.8)	34 (68.0)	16 (32.0)
Male	38 (43.2)	25 (65.8)	13 (34.2)
Age in years: median (IQR)	34.5 (27.5,44.0)	38.2 (12.2)	33 (24,42)
HIV status			
Negative	29 (32.9)	–	–
Positive	59 (67.1)	–	–
ART status in months			
1	30 (34.1)	30 (50.8)	–
2	29 (33.0)	29 (49.2)	–
CD4 count: median (IQR)	402 (231,596)	402 (231,596)	–
Viral load			
<50	49 (62.8)	49 (62.8)	–
50+	10 (100)	10 (100)	–
DMFT: mean (SD)	5.2 (4.9)	4.9 (4.6)	5.9 (5.5)
DMFT category			
Healthy (0)	11 (12.5)	8 (72.7)	3 (27.3)
Low (1–3)	32 (36.4)	20 (62.5)	12 (37.5)
Medium (4–6)	21 (23.9)	17 (80.9)	4 (19.1)
High (7–13)	16 (18.2)	10 (62.5)	6 (37.5)
Extremely high (≥14)	8 (9.0)	4 (50.0)	4 (50.0)
Periodontal status			
Gingivitis	51 (57.9)	31 (60.8)	20 (39.2)
Periodontitis	37 (42.1)	28 (75.7)	9 (24.3)
Saliva flow rate: mean (SD)	0.9 (0.5)	0.8 (0.5)	1.0 (0.5)

### Oral microbial community structure

**Figure 2:**
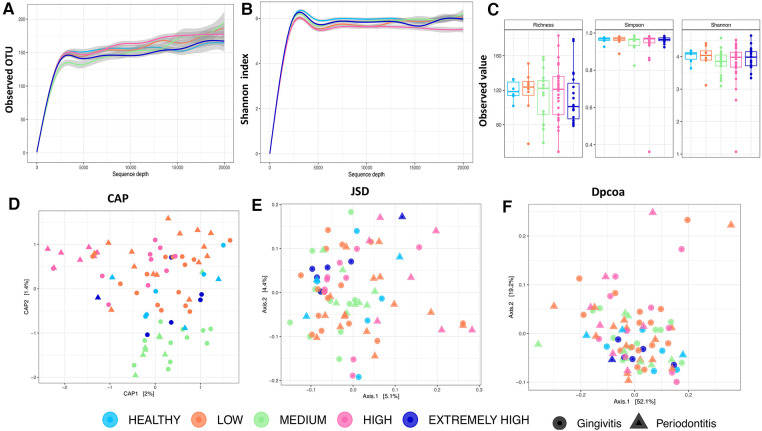

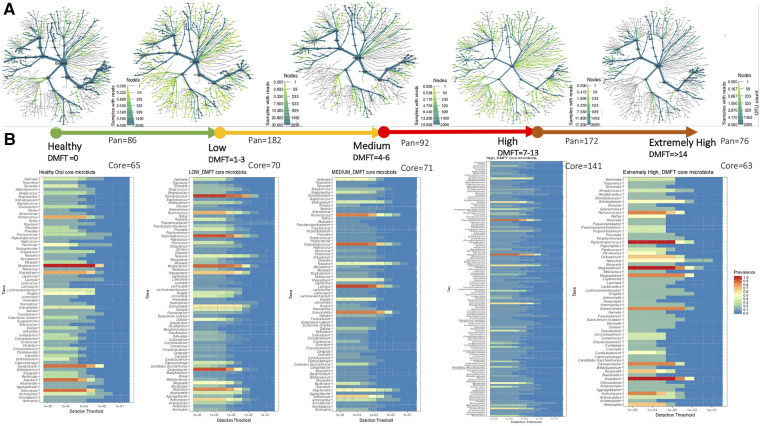
(**A**) shows the microbial community structure across the DMFT categories. Panel (**A**–**C**) show alpha diversity characteristics i.e., the rarefaction curves of observed OTU and Shannon index colored by DMFT category. Panel (**C**) show a boxplot of alpha diversity indices at a minimum sequence depth set at 3000. Panel (**D**–**F**) shows Beta diversity indices. The shapes represent dental caries and periodontal health status (gingivitis/periodontitis). (**B**) Shows the pan(**A**) and core(**B**) oral microbiota along the DMFT index highlighted in arrows colored in order of severity. Panel (**A,B**) represent genera abundance and prevalence respectively.

A total of 2,601,254 high quality *16S rRNA* sequences were recovered from the 88 participants. Samples with the highest and lowest sequence count came from HIV negative and HIV positive participants, respectively (Supplementary Figure S1). When the sequences were filtered to a depth of 3,000, we retained 80 participants (Supplementary Figure S1) from whom 2,093 OTUs were generated with a median frequency of 14,351. There was no statistically significant difference in DMFT categories with regard to alpha diversity indices (Figure 2A, panel A–C) but a statistically significant association (*p* < 0.005) was observed between DMFT categories and beta diversity indices such as the unweighted Unifrac and Bray-Curtis distances (Figure 2A, panel D–F & Supplementary Figure S2). There was some clustering along the DMFT index specifically separating low and medium categories (Figure 2A, panel D).

### Oral microbial composition

**Figure 3:**
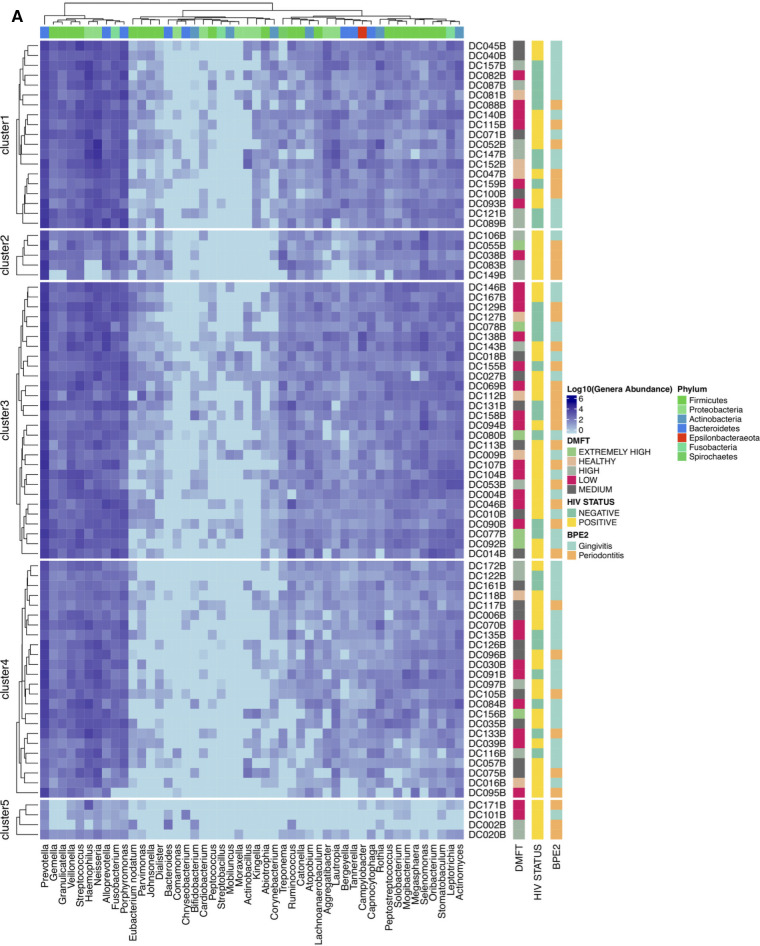

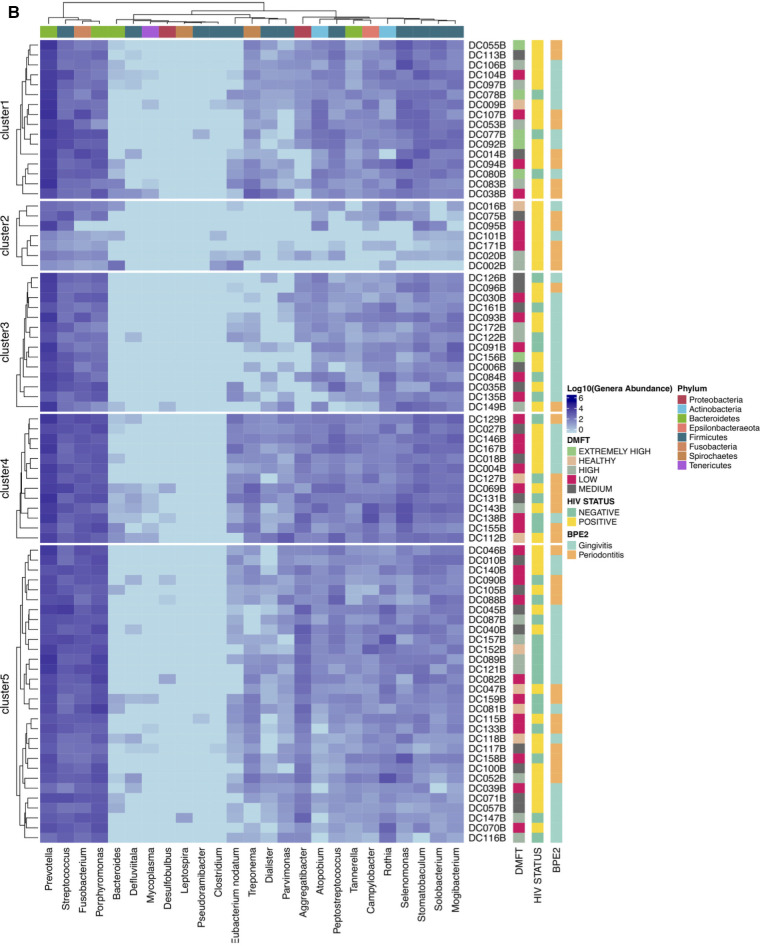

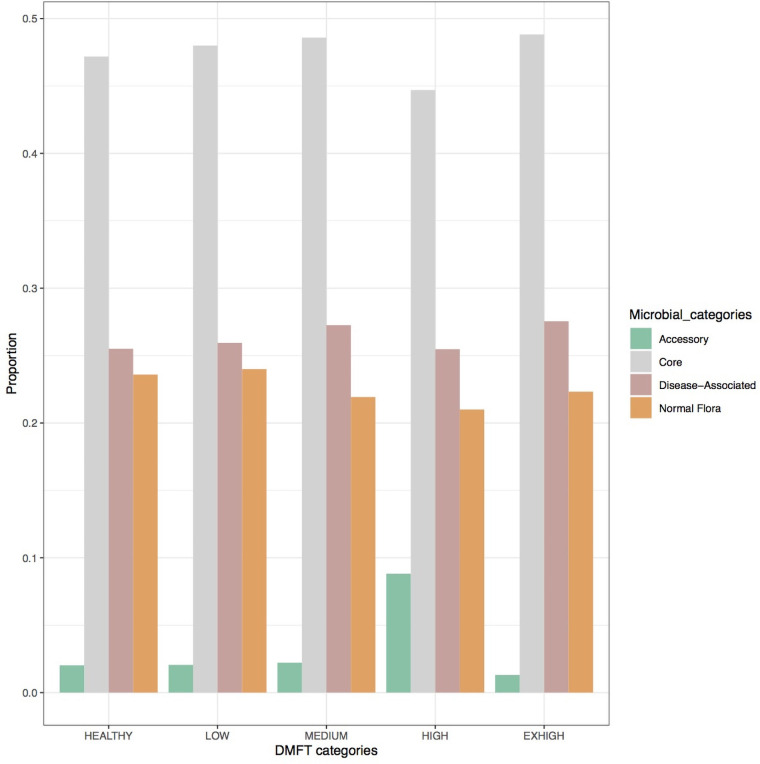
Shows the different microbial components tracked along the DMFT. Panel (**A**) is the DMFT core, which is the genera present at all stages of the DMFT, Panel (**B**) shows the genera previously associated with oral disease characterized by patient ID, HIV status and type of oral disease. Panel (**C**) shows the different proportions of microbial groups along the DMFT categories.

The above association was further explored at a taxonomic level i.e., core and accessory microbiota prevalence and abundance (Figure 2B, panel A,B). The 2,093 OTUs clustered into 21 phyla and 239 genera. *Firmicutes* (33.3%), *Bacteroidetes* (32.3%) and *Proteobacteria* (16%) were the dominant phyla, however, at genus level *Prevotella* (12%), *Porphyromonas* (3.7%), *Leptotrichia* (3.4%), *Selenomonas* (3.4%) were the most predominant (Supplementary Figure S3). Interestingly we detected the genus *Mycobacterium* DNA from three HIV positive patients and a potential linear relation with CD4 T cell count (Supplementary Figure S4). We also noted that clusters 2 & 5 (Figures 3A, B), were exclusively composed of HIV positive participants; cluster 2 was characterized by a low abundance of genera associated with oral disease while cluster 5 by those that are part of the core.

### Apparently-healthy individuals (DMFT = 0)

Among the eleven apparently healthy individuals the core and pan microbiota size was 65 and 86 genera respectively, i.e., an accessory microbiota of 21 genera. The most abundant core genera included; *Ruminococcus*, *Mogibacterium*, *Megasphaera*, *Campylobacter*, *Atopobium* and *Actinomyces* (Figure 2B, panel B). The core, normal flora and genera associated with dental caries accounted for 49%, 23% and 27% respectively while the accessory microbiota accounted for 1% (Figure 3C). Of the 65 genera observed among apparently healthy individuals, 49 were shared across the different DMFT categories, hence forth referred to as the DMFT core.

### Low DMFT

**Figure 4:**
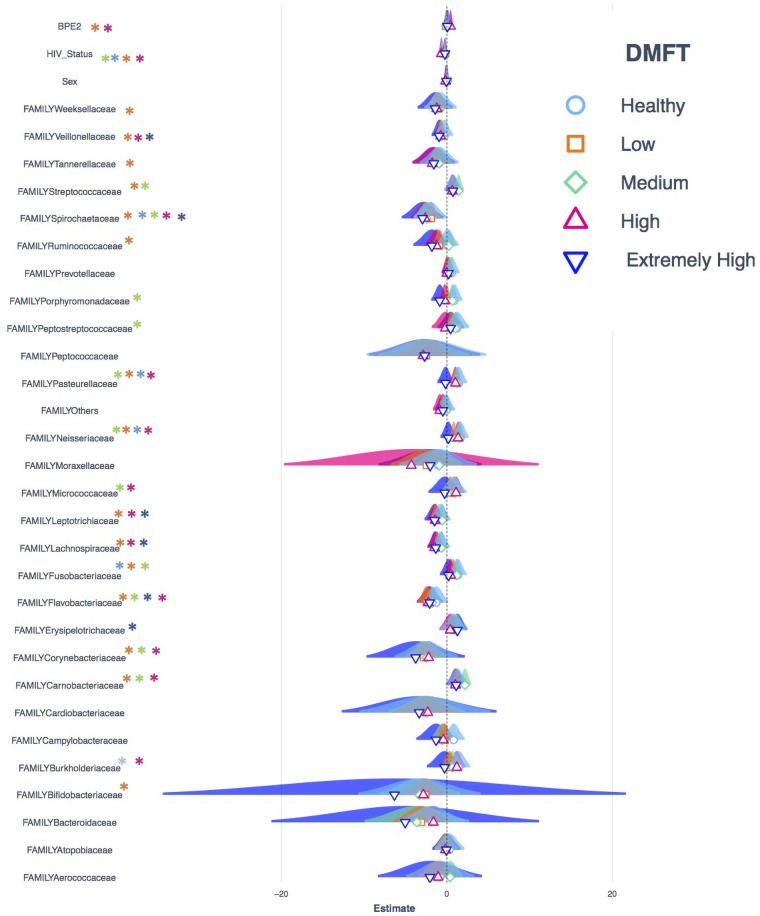
Shows the Poisson regression model of taxonomic abundance and clinical attributes, a comparison of models generated from datasets that represent each DMFT category. The Asterix colors shows in which dataset a variable is statistically significant.

The transition from apparently healthy to low DMFT category was characterized by a slightly higher proportion of the core but the proportion of the accessory microbiota at this stage remained relatively unchanged. This was associated with a greater abundance of *Stomatobaculum*, *Peptostreptococcus* and a lower abundance of *Atopobium* and *Actinomyces* (Figure 2B, panel B). Here the families with significant differences in abundance included *Weeksellacae*, *Veillonellaceae*, *Streptococaccae*, and *Ruminoccocaea* (Figure 4).

### Medium and high DMFT

The medium DMFT category was distinctly separated by microbial clustering (Figure 2A, panel D), and was also characterized by a slightly higher proportion of the core microbiota. The core, normal flora and oral disease associated genera accounted for oral microbial richness of 48%, 22% and 27% respectively (Figure 3C). This difference was associated with an enrichment of *Ruminococcus*, *Peptospteptococcus* and *Lautropia* (Figure 2B, panel B). Participants in the high DMFT category and with periodontitis carried significantly more microbial biomass (Figure 4 and Supplementary Figure S6). Indeed, at this stage the accessory microbes accounted for 9% of the microbiota in the oral cavity (Figure 3C). However, families whose abundance was significantly different primarily belonged to the core; *Carnobacteriaceae*, *Neisseriaceae*, and *Micrococcaceae*.

### Extremely high DMFT

In comparison with the apparently healthy and low DMFT categories, this stage was characterized by a slightly higher abundance of *Solobacterium*, *Oribacterium*, *Neisseria*, *Granulicatella*, *Atopobium*, *Abiotrophia* and a lower abundance of *Campylobacter* (Figure 2B, panel B). On the other hand, the proportion of the accessory microbiota fell at this stage (Figure 3C).

### Changes in community entropy

**Figure 5:**
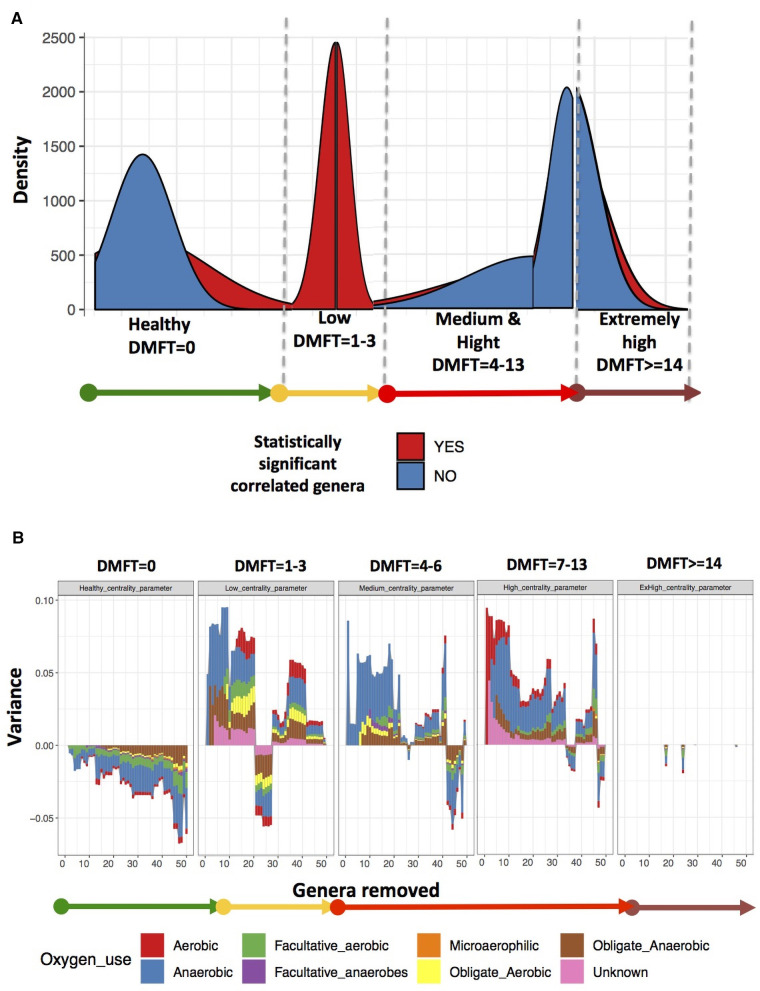
Shows the oral microbial co-occurrence network characteristics. Panel (**A**) shows the changes in microbial community entropy, Panel (**B**) shows variance and the genera to which the variance is attributable.

There was a characteristic difference in entropy i.e., the proportion of genus pairwise association that are statistically significant (Figure 5A). The low DMFT category was associated with a much lower entropy i.e., ∼99% of pairwise correlations were statistically significant i.e., a change from random to non-random (Figure 5A and Supplementary Figure S7). As the severity of dental caries progressed, entropy returned i.e., co-occurrence clusters gradually changed from non-random to random.

### Genera associated changes in variance

We noted among the apparently healthy participants a difference in variance of up to 5%, most of which was attributable to core genera (Figure 5B). This implied that the most influential genera at this stage belonged to core (see full list of genera identified as influential in Supplementary data). In the low DMFT category, the difference in variance was up to 10%, attributable to 20 genera, most of which belonged to the accessory oral microbiota. These included; *Treponema*, *Desulfoplanes*, *Desulfosporosinus*, *Sphaerochaeta*, *Arenimonas*, and *Microbacterium* among others. Furthermore, these belonged to the families with statistically significant differences in abundance in this category. The medium and high DMFT categories were also characterized by differences in variance of as high as 10%, and we observed that the composition of influential genera changed from aerobic to anaerobic. Unlike the low DMFT category, here 98% of the influential genera belonged to the core (see full list of genera identified as influential in Supplementary data). The extremely high DMFT category was characterized by lower difference in variance, and here too almost all the influential genera were members of the core.

## Discussion

Even though the pathogenesis of dental caries has for long been linked to certain microorganisms (3), its routine diagnosis is almost exclusively macroscopic. This is mainly because microbial culture-based testing is laborious and complicated (33), which delays comprehensive and timely delivery of clinical results. However, with the advent of culture-independent NGS approaches like *16S rRNA* amplicon sequencing (34), we can narrow this gap in the developing countries where HIV may be disproportionately driving the occurrence of oral disease. In this study, we used amplicon sequencing data to characterize the oral microbiota along the routinely used diagnostic DMFT index in HIV positive and negative participants.

### Changes in oral microbiota along the DMFT index

#### Apparently healthy individuals

Microbiota is integral to the oral cavity and plays a critical role in maintaining its integrity (3). In apparently healthy individuals, this manifests as stability in structure and composition (33, 35). In this study, we observed a relatively stable core size of 49 genera across all DMFT categories. The notion of a microbiome core is commonly investigated as a proxy for resident microbes of any given microbiome (36). Among apparently healthy individuals, we observed genera such as *Streptococcus*, *Staphylococcus*, *Corynebacterium*, *Veillonella*, *Granulicatella* and *Gemella*, most of which have been reported as normal flora of the oral cavity (37). The proportion of accessory microbiota, referred to as invaders, was at its lowest among the apparently healthy individuals. This lends support to the notion that the normal flora inhibit invaders through the production of substances such as fatty acids, peroxides and bacteriocins in order to maintain its integrity in healthy individuals (38). This concerted activity has also been characterised among functional communities (39), which in this study are analogous to co-occurrence communities. Indeed, among healthy individuals we observed that the co-occurrence communities were dominated by members of the core such as *Johnsonella*, *Rikenella*, *Porphyromonas*, *Alloprevotella, Tannerella*, *Fusobacterium* and others. These organisms are predominantly anaerobic (28) probably due to microbial succession that occurs during the formation of dental plaque (40).

#### Low DMFT

The onset of pathology is widely associated with oral microbiome dysbiosis and the forces that modulate this, are similar to selection forces i.e., changes in fitness, growth and reproduction of microbes manifested as fluxes in prevalence and abundance (3). In the midst of such forces, the core size remained stable with a slightly higher proportion of accessory microbes, particularly anaerobic lactate fermenters like *Sharpea*, *Lawsonella* and *Olsenella*. These microorganisms have been implicated in endodontic infections and acute apical abscesses (41, 42). There was considerably higher abundance of genera such as *Stomatobaculum*, *Peptostreptococus*, *Campylobacter* and *Mogibacterium* which have been associated with subgingival pathology (43). In this category, we noted that individuals with gingivitis carried significantly more microbes, and families such as *Weeksellacae*, *Veillonellaceae*, *Streptococcaceae* and *Ruminococcacaea* accounted for the major difference in abundance. These organisms have been associated with dental caries (28, 44) and labial abscesses (45) and it is most probable that changes in the local environment and microbial interactions favor their growth. We noted the absence of genera such as *Roseburia*, which is associated with health elsewhere (46), and its absence here probably supports the onset of pathology. The development of dental caries can also be viewed as onset of a constraint to the normal microbial community interaction. In this study, this constraint was detectable as a difference in entropy, that is, 99% of the pairwise correlations were statistically significant. In other words, the probability that the associations observed are occurring randomly is very low, which suggests an overwhelming constraint. This difference in entropy is also associated with up to a 10% variation, attributable to genera such as *Lachnospira*, *Treponema*, *Aggregatibacter*, *Corynebacteria* and *Bifidobacteria* whose roles in pathogenesis of oral diseases are well documented (47–50). However, some of the influential genera such as *Ferruginibacter* and *Sphaerochaeta* are part of the accessory microbiota.

#### Medium and high DMFT

The proportion of the accessory microbiota was highest in the high DMFT category compared to all the other DMFT categories. It is well established that caries development is associated with the interaction of different microorganisms within a cariogenic biofilm, which change as caries progresses (51). Here, there was a greater abundance of core members, *Rothia* and *Mogibacterium*, which are associated with dental caries (52) and periodontal disease (52, 53), and *Lautropia*, which has been isolated from oral cavities of HIV infected children (54) but is not associated with oral disease (54, 55). However, as severity of dental caries changed, the resident microbes seemed overwhelmed, which was seen as a surge in the accessory microbiota. Microbiota in the high DMFT category appeared to have gone through significant remodeling because most of the invading population in the medium category became established as members of the core. Moreover, it is in these two categories and at low DMFT that we observed the highest proportion of the least biologically characterized organisms.

#### Extremely high DMFT

Although this category was characterized based on only six individuals, nonetheless, we observed a greater abundance of microorganisms although the accessory size was smaller. The core was dominated by genera such as *Peptostreptoccus*, *Mogibacterium* and *Atopobium* which have been implicated in chronic oral pathology (56). We also observed a difference in variation and genera such as *Solobacterium*, *Ruminococcus*, *Neisseria*, *Campylobacter*, *Atopobium* and *Abitrophia* have the largest difference in abundance.

#### Microbiota along the DMFT index

**Figure 6:**
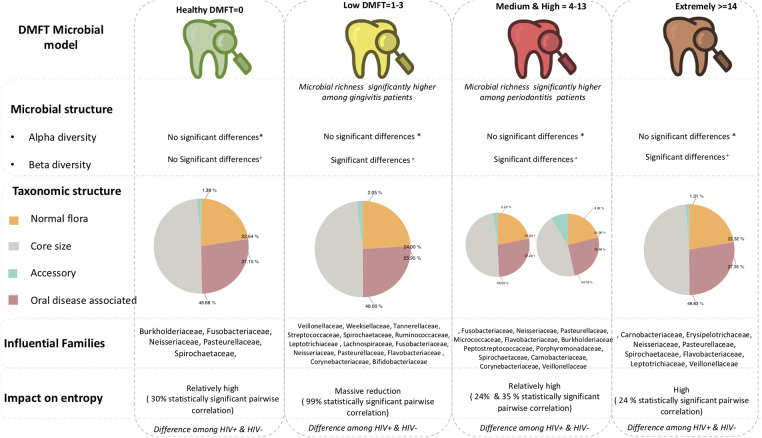
Shows the proposed framework for investigate oral microbial characteristics in LMICs.

From these findings, we propose a microbial model of the DMFT index (Figure 6) as a foundation for oral clinical metagenomics in resource limited settings. (1) Microbial structure; alpha diversity index does not discriminate along the DMFT index but beta diversity does. (2) Taxonomic composition; the DMFT core genera are approximately twice the normal flora in most categories of the DMFT index. In addition, at dental caries onset/low DMFT, the number of genera whose abundance is significantly different belong to the core, this gradually changes to the accessory in the medium and high DMFT categories. (3) Entropy; dental caries onset creates a microbiome wide constraint detectable as a difference in entropy. Almost all genera-pairwise correlations are statistically significant at this stage interpreted as a reduction in microbial entropy. (4) Periodontal health status; we noted a significant difference in microbial abundance i.e., the low DMFT category best represents gingivitis while the high DMFT category represents periodontitis. With this information, a testable hypothesis about pathobiology, probiotic supplementation and treatment can be generated and tested to improve dental caries management in people living with HIV.

### Limitation of the study

In this study, we did not have equal numbers of patients in each group i.e., 11, 32, 21, 18 and 6 for healthy, low, medium, high and extremely high DMFT categories. This has the potential of increasing the level of uncertainty for the estimates made for groups with fewer individuals. However, we observed a considerable level of consistency in microbes in the different categories, therefore, the impact of sample size per group is likely to be limited.

## Conclusions

In conclusion, we characterized the oral microbiota dynamics along the DMFT index in HIV positive and negative individuals. Although the microbial characteristics did not offer a categorical output of the DMFT index, they provided the following insights; (a) the low DMFT category showed significant differences in core genera abundance, (b) the high DMFT category was associated with a higher proportion of accessory microbiota, (c) the influential genera in apparently healthy patients were predominantly core members whereas it was the accessory in the medium and high DMFT categories, (d) low DMFT category was associated with a massive reduction in oral microbial entropy. Therefore, using this information, we have proposed a microbial framework for characterizing the DMFT index to better understand dental caries, and serve as a foundation for improving dental caries diagnosis and management in people living with HIV in resource limited settings.

## Data Availability

The sequences generated for this study have been deposited in the Sequence Read Archive of NCBI (Project Number: PRJNA627249). All other data is contained in the main manuscript and supplemental files.
