# Supplementary figures and images for: Microbial characteristics of dental caries in HIV positive individuals
Source: Front Oral Health. 2022 Sep 21;3:1004930. doi: 10.3389/froh.2022.1004930 (PMC9533146; doi:10.3389/froh.2022.1004930)

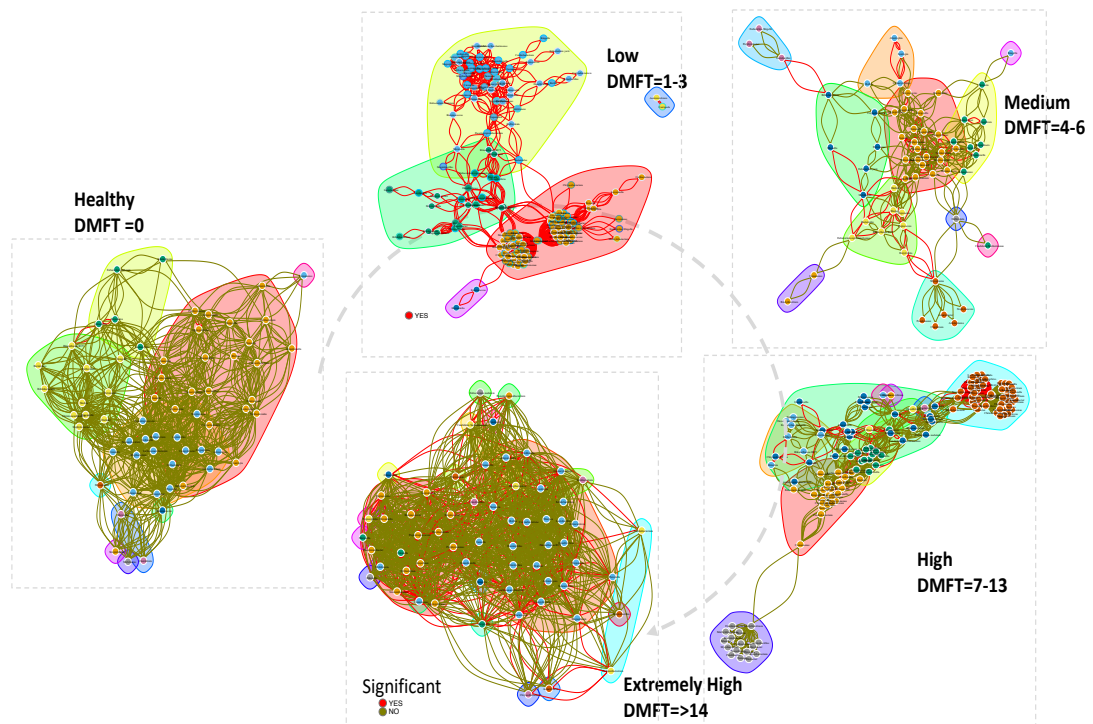

Supplement: Supplementary file 2 [file DataSheet1.pdf]

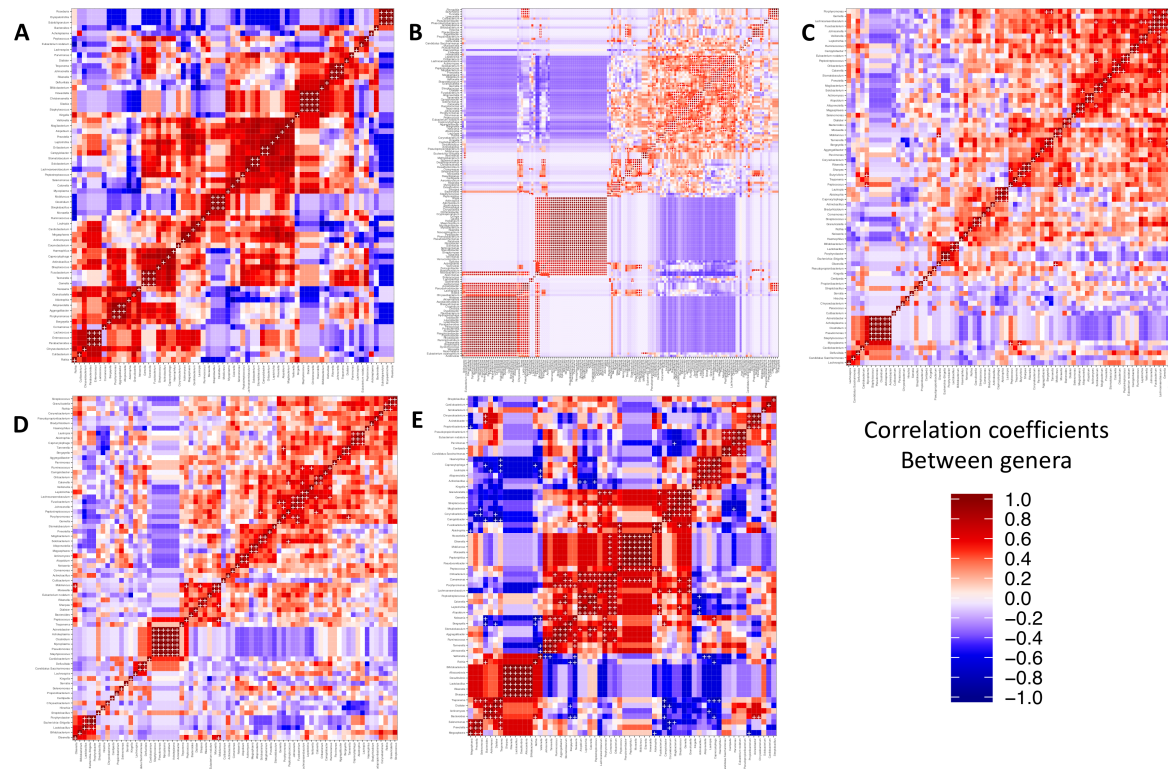

Supplement: Supplementary file 4 [file DataSheet3.pdf]

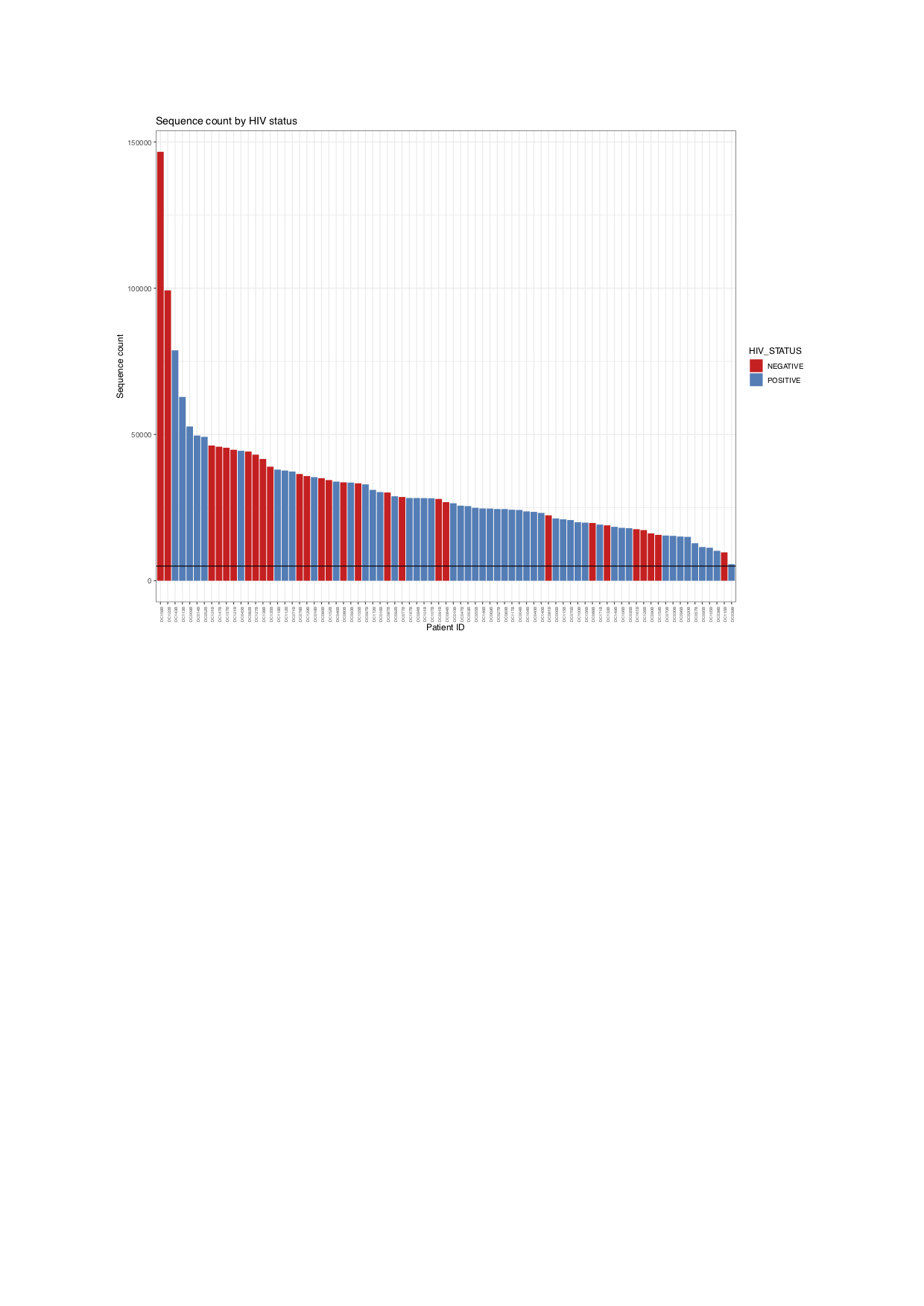

Supplement: Supplementary file 5 [file Image1.jpeg]

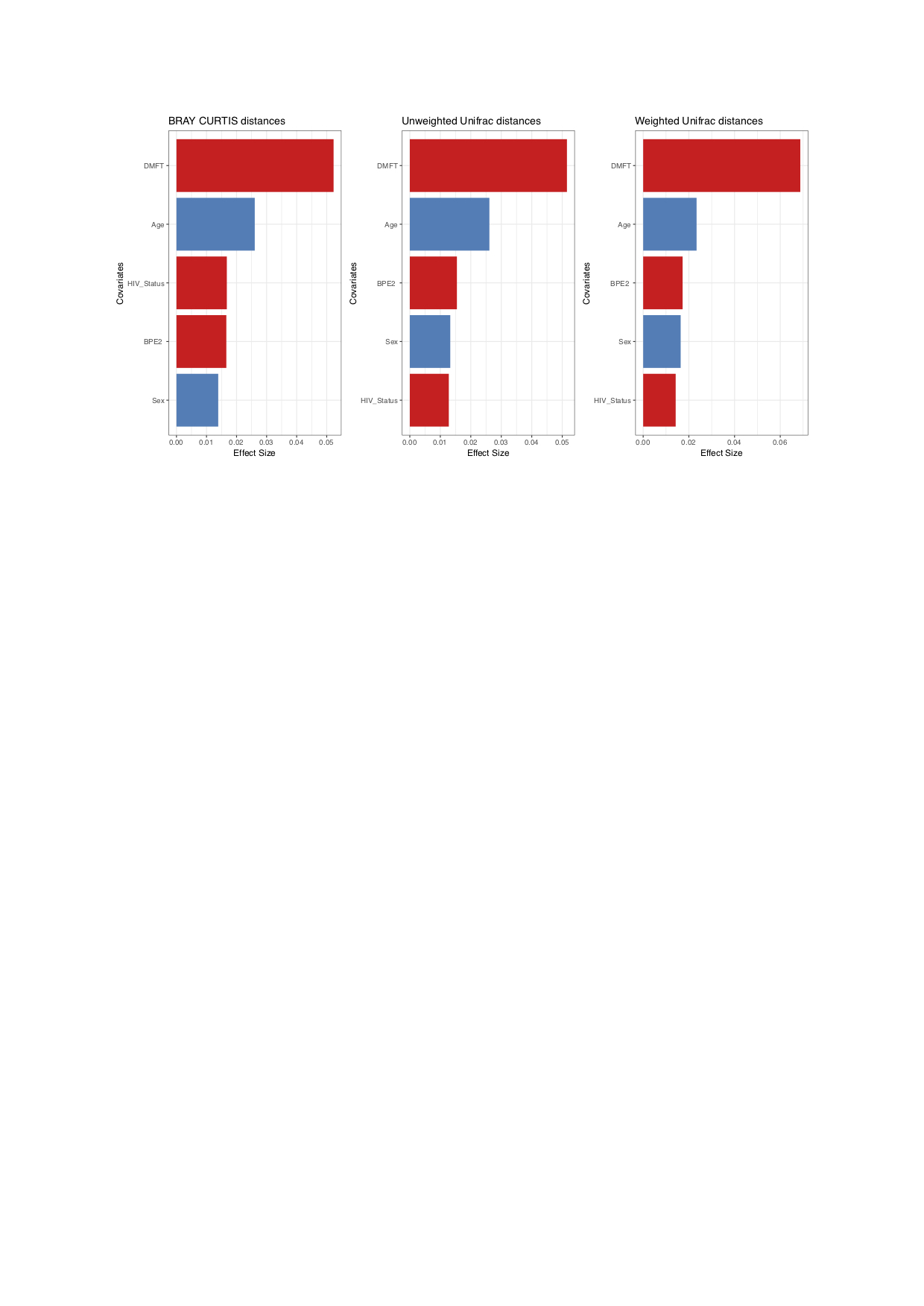

Supplement: Supplementary file 6 [file Image2.jpeg]

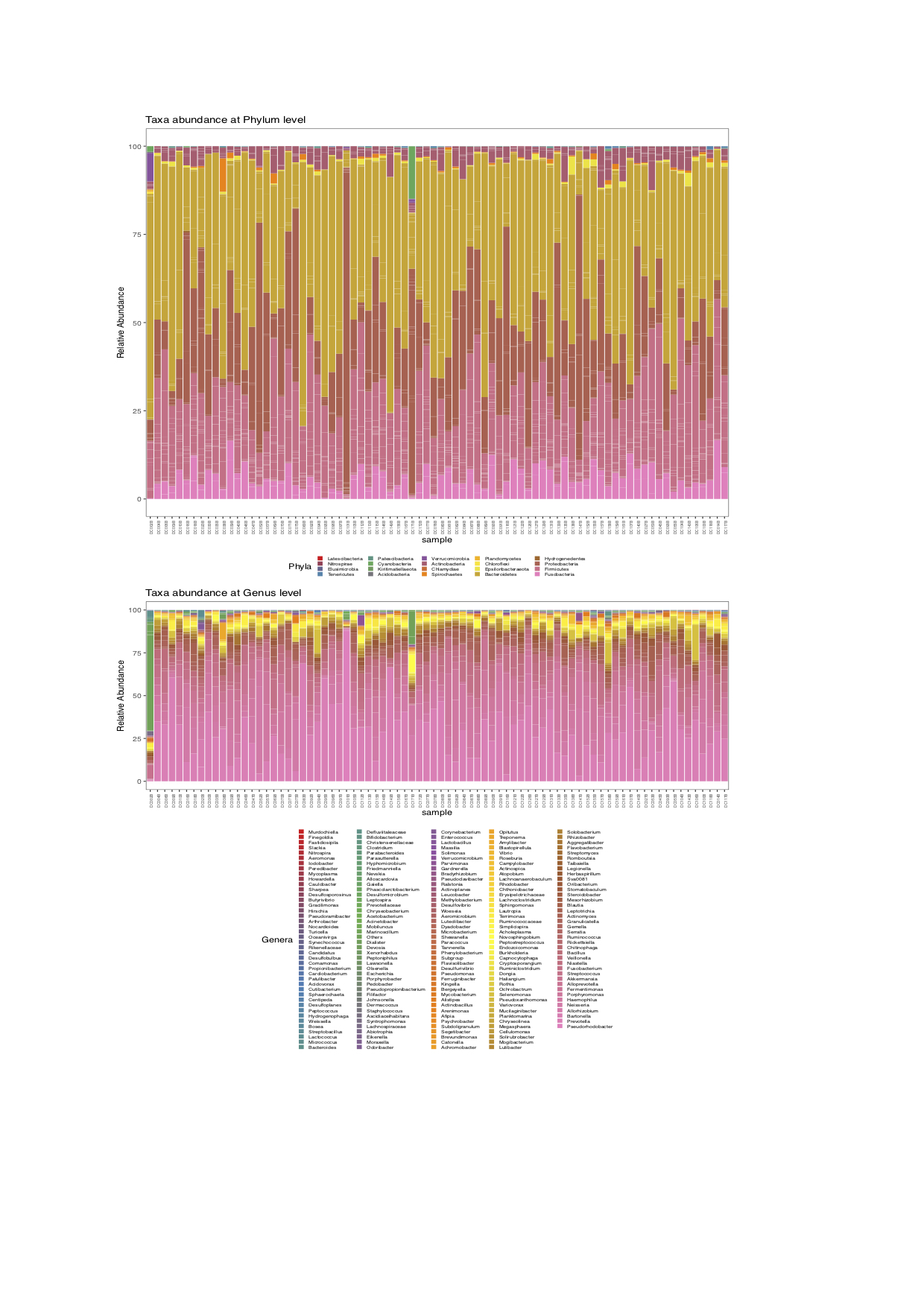

Supplement: Supplementary file 7 [file Image3.jpeg]

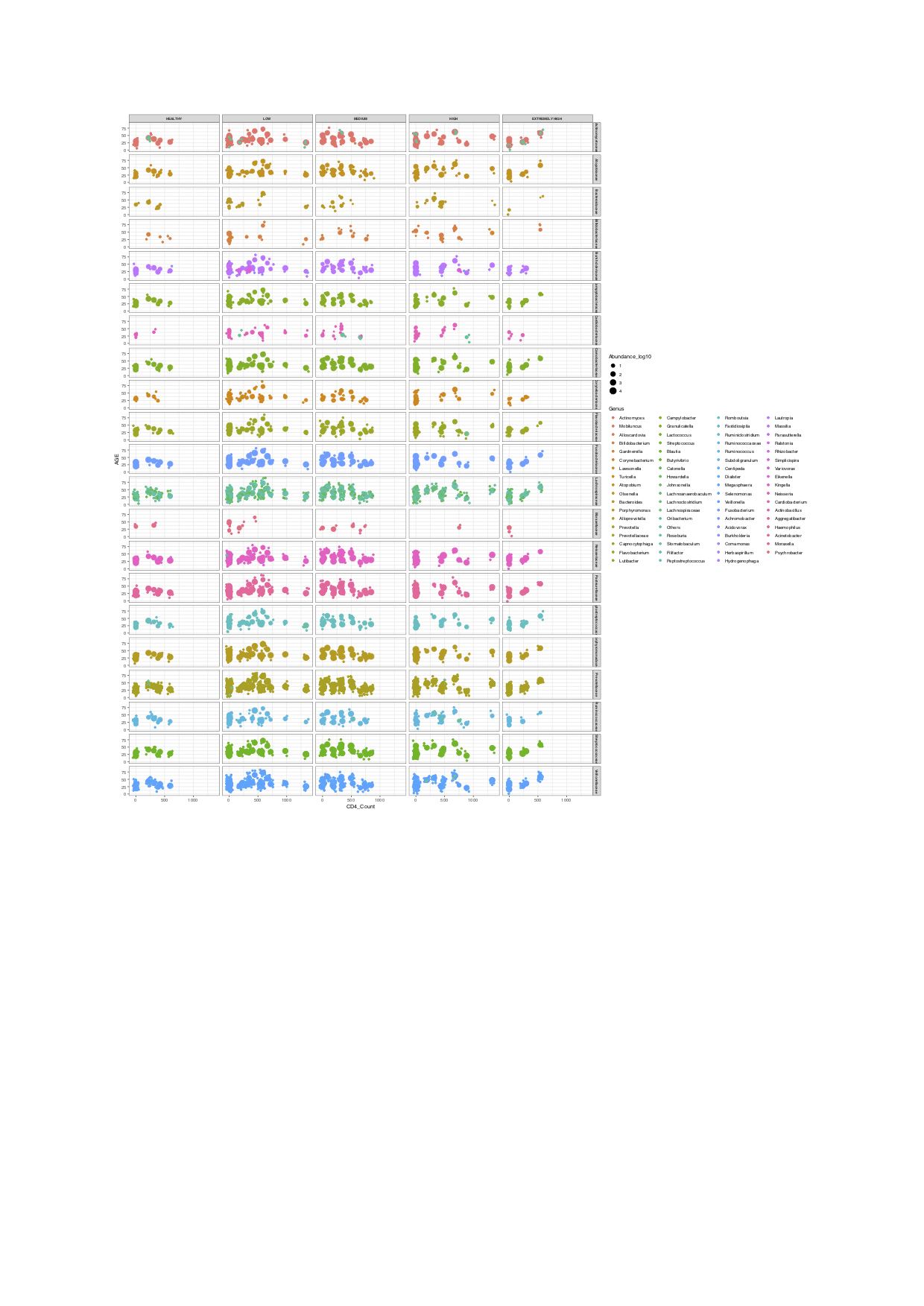

Supplement: Supplementary file 8 [file Image4.jpeg]

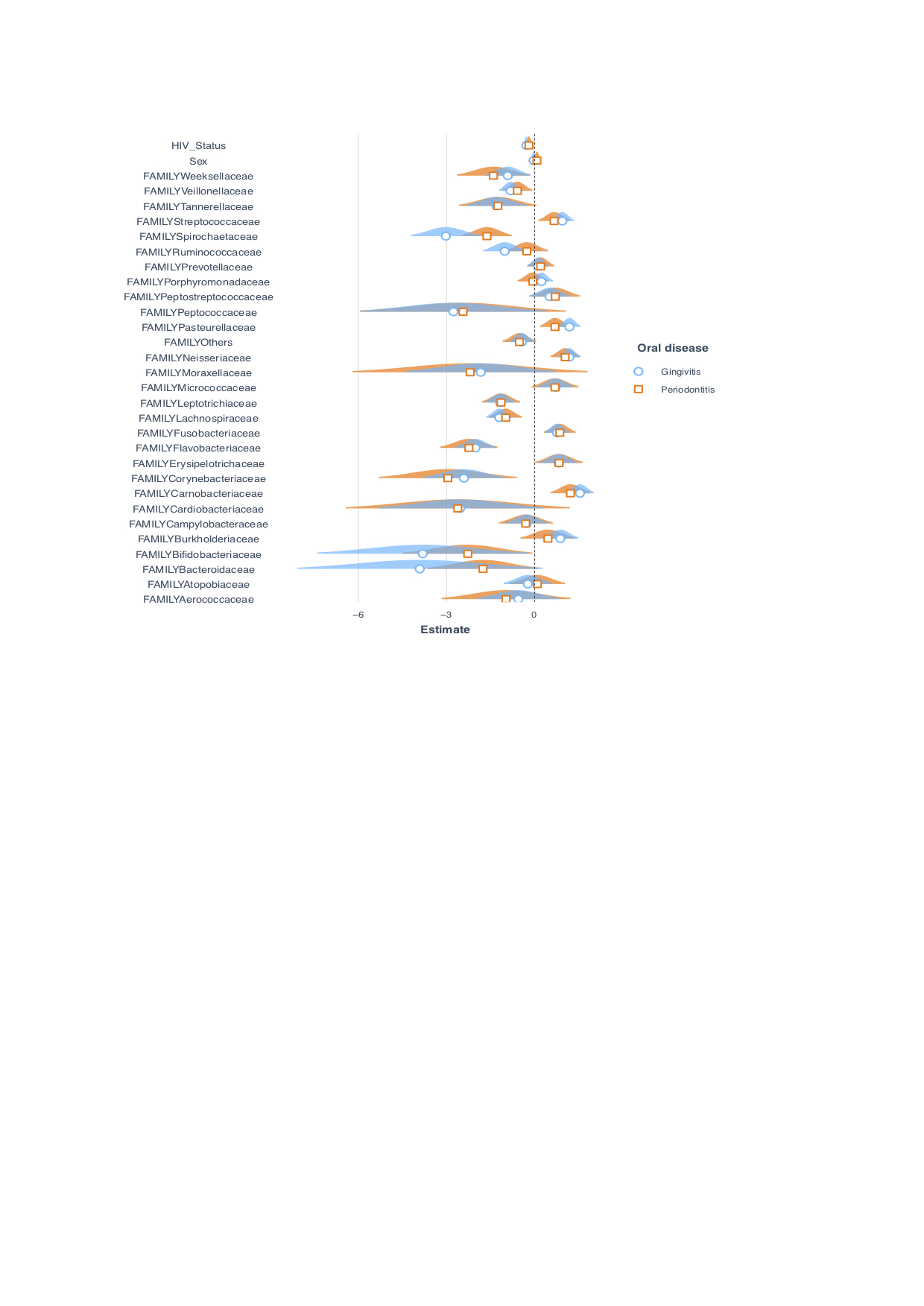

Supplement: Supplementary file 9 [file Image5.jpeg]
